# A phase I clinical trial utilizing autologous tumor-infiltrating lymphocytes in patients with primary hepatocellular carcinoma

**DOI:** 10.18632/oncotarget.5463

**Published:** 2015-10-20

**Authors:** Shan-Shan Jiang, Yan Tang, Yao-Jun Zhang, De-Sheng Weng, Zhong-Guo Zhou, Ke Pan, Qiu-Zhong Pan, Qi-Jing Wang, Qing Liu, Jia He, Jing-Jing Zhao, Jiang Li, Min-Shan Chen, Alfred E. Chang, Qiao Li, Jian-Chuan Xia

**Affiliations:** ^1^ Department of Biotherapy, Sun Yat-sen University Cancer Center, State Key Laboratory of Oncology in South China, Collaborative Innovation Center for Cancer Medicine, Guangzhou, China; ^2^ Department of Hepatobiliary Oncology, Sun Yat-sen University Cancer Center, Guangzhou, China; ^3^ University of Michigan Comprehensive Cancer Center, Ann Arbor, Michigan, USA

**Keywords:** primary hepatocellular carcinoma, autologous tumor-infiltrating lymphocytes, adoptive cell therapy

## Abstract

This report describes an ongoing Phase I clinical trial testing the safety of adoptive cell therapy (ACT) using autologous tumor-infiltrating lymphocytes (TIL) in patients with primary hepatocellular carcinoma (HCC). Fifteen HCC patients were treated with their activated and expanded TILs following tumor resection. From a total of 17 patients with HCC, TIL were successfully expanded from 15 patients (88%), whereas two patients showed minimal or no expansion of TIL. Transient increase in the frequency of T cells was observed after adoptive transfer who was found only associated with grade I flu-like symptoms and malaise. After a median follow-up of 14 months, 15 patients (100%) were alive; and 12 patients (80%) showed no evidence of disease, 3 patients (patient 1,11,12) had tumor recurrence. The time to the diagnosis of tumor recurrence following therapy ranged from 105 to 261 days. These results indicate that immunotherapy with activated and expanded autologous TIL could be successfully performed with low toxicity, thus would serve as a novel treatment modality for patients with HCC.

## INTRODUCTION

Hepatocellular carcinoma (HCC) is one of the most prevalent and lethal malignancies worldwide. It is the fifth most common cancer and ranks as the third leading cause of cancer-related deaths. In China, current treatment options for early-stage HCC include surgery, transarterial chemoembolization (TACE) and molecular targeted therapy. However, the overall outcome of such efforts towards HCC is disappointing, with only 10–20% of tumors resectable at the time of diagnosis, and the five-year survival is poor compared with other gastrointestinal malignancies [1, 2].

Previously, patients with advanced HCC did not have very much therapeutic options. A major milestone in the treatment for this disease was the clinical use of sorafenib. Sorafenib, a multikinase inhibitor targeting the Raf serine/threonine kinases and the VEGF receptor 1–3 (VEGFR1-3), PDGF receptor (PDGFR)-b, c-Kit, fms-like tyrosine kinase-3 (FLT-3) and p38 tyrosine kinases [3], was the first approved molecule-targeted agent that demonstrated survival benefits in patients with advanced HCC in 2007 [4, 5]. It inhibits tumor-cell proliferation and tumor angiogenesis and increases the rate of apoptosis in a wide range of tumor models [6, 7]. The clinical success of sorafenib occurred when two pivotal studies, the SHARP and Asian Pacific trials, showed survival benefits over best supportive care [8, 9]. These results have led to the approval of sorafenib by regulatory agencies as the first systemic treatment in the advanced HCC and have served as a major impetus to recent research in the field.

Immunotherapy is emerging as a new treatment stratage for tumors, which has been cosidered as the fourth treatment option following surgery, chemotherapy and radiotherapy. Immunotherapy has proved to play an important role in the comprehensive treatment of tumors [10]. For example, the success of immunotherapy in advanced prostate cancer suggested its significant role in cancer treatment [11]. Adoptive cell therapy (ACT) is a highly personalized form of passive immune therapy based on the infusion of autologous T cells that are activated and expanded *ex vivo* [12]. Tumor-infiltrating lymphocytes (TILs) are a type of white blood cells found in tumor, and are implicated to be tumor-reactive. The presence of TIL in tumors is often associated with improved clinical outcomes [13, 14]. TILs are frequently found in tumor, suggesting tumor immunogenicity and immune response to tumor antigens in the host. These antigens distinguish the tumor from healthy cells, thereby providing an immunological stimulus [15]. Clinical-grade TIL generation for human therapy is a biphasic process, consisting of initial expansion in IL-2-containing medium of TILs obtained from tumor fragments or digests (pre-rapid expansion protocol [REP] phase). Following a standard 14-day REP, the TILs are activated with anti-CD3 antibody and irradiated allogeneic peripheral blood mononuclear cells (PBMCs) [16]. Rosenberg and colleagues previously described TIL treatment of patients with advanced melanoma in three clinical trials at the NIH Clinical Center. Improved patient outcome was observed when TILs were used compared with lymphokine activated killer cells (LAKs) [17]. Recently, phase II trials have illustrated strong potential of TIL in malignancy therapy, with promising cure rates of 20–40% [18]. Previous studies have demonstrated remarkable correlations between tumor prognosis and the number as well as the component of TILs in HCC [19, 20]. Therefore, isolating, stimulating and amplifying TIL from HCC could potentially represent a novel immunotherapy against HCC.

## RESULTS

### Patient characteristics

Fifteen patients were enrolled in the study, and all patients presented with HCC without distant organ metastasis at diagnosis. Among the 15 patients, 14 were male and 1 was female. The average age was 46.7 years (range, 31–66 years). Three patients had a performance status (PS) of 1; all others had a PS of 0. All fifteen patients had hepatic B virus infection, underwent radical resection prior to receiving TIL therapy and were followed-up to monitor toxicity. The median follow-up period was 14 months (range, 12–16 months).

### *In vitro* expansion and characterization of TIL

TILs were successfully generated from all 15 patients with HCC recruited for the study. TILs from all patients were activated and expanded within 27.9 ± 3.05 days (range, 23–33 days) using the method described in the Materials. TIL cultures were either cryopreserved or used directly for large-scale expansion. All cultures were expanded to treatment levels. TILs were administered at an average of 27.7 days after surgery.

To evaluate basic TIL characteristics, fluorescence-activated cell sorting (FACS) analysis was performed and IFN-γ expression was assessed after PFA stimulation. The T cells generated for therapy contained a high frequency of CD3^+^ lymphocytes, ranging from 86.2% to 99.2% (average, 95.8%). These CD3^+^ populations contained a number of heterogeneous CD8^+^ T cells, ranging from 20.7% to 82.3% (average, 42.7%), and a number of CD4^+^ T cells, ranging from 18% to 80.5% (average, 50.5%), and a number of CD56^+^ T cells, ranging from 2.2% to 36.4% (average, 14%). Futhermore, The average CD8^+^CD27^+^ frequency is ranging from 9.2% to 59.8%(average, 25.3%) (Table 1).

**Table 1:** Characteristics of infused TILs

Patients	Infused TILs (%)					CD8^+^ cells		
	CD3	CD4	CD8	CD56	CD8^+^CD27^+^	IFN-γ	TNF-α	IL-4
01	97.8	63.5	31.7	11.1	22.4	18.5	26.3	4.5
02	99.2	80.5	20.7	7.3	15.9	31.3	32.6	5.4
03	99.2	56	43.2	13	36.6	26.8	20.7	6.1
04	98.4	18	80.3	5.7	13.7	70.4	62.6	1.1
05	94.8	48.3	44	15.5	19.7	34.6	37.3	4.1
06	90.8	79	11.7	3.7	25.7	11.6	10.8	2.5
07	94.7	78	16.5	10.6	12.1	13.5	12.6	7.8
08	98.7	16.7	82.3	2.2	26.6	79.8	51.4	5.3
09	98.7	41.6	49.2	30.4	51.8	46.8	33	6
10	96.9	54.4	42.1	20	25.3	66.1	37.6	2.6
11	92.1	49.6	30.2	23.8	9.2	23.4	19.7	1.5
12	96	62.1	30.7	6.1	13.4	35.7	42.2	2.8
13	86.2	31.2	44.6	36.4	25.3	43.6	36.2	28.5
14	96.8	28.4	66.3	13.2	59.8	38.5	34.9	8.2
15	97.2	50.3	46.3	13.2	22	40.8	32.8	10.5
Total average	95.8 ± 3.66	50.5 ± 20.6	42.7 ± 20.9	14 ± 9.8	25.3 ± 14.3	38.76	32.7	5.12

To determine the immune response of TIL after expansion, the cytokine profiles were evaluated, including type 1 cytokines IFN-γ and TNF–α and type 2 cytokine IL-4, in TIL cultures from 15 HCC patients treated with PMA/ionomycin (Table 1). The TIL cultures contained a high percentage of CD8^+^ IFN-γ producing T cells (38.76% ± 20.3%) and CD8^+^ TNF-α producing T cells (32.7% ± 13.7%), and a low percentage of CD8^+^ IL-4 producing T cells (5.1% ± 2.8%). These results suggest that the TIL cultures genarated from HCC patients were prioritized to secret type 1 cytokines after T cell receptor stimulation in this study.

In the present research, there are striking differences in the relative proportions of CD4, CD8 and CD56 cells. We have performed the correlation analysis for proportions of CD4, CD8, and CD56 cells with patient's tumor type, cytokine production, and toxicity. The results are shown in Table 2. In the correlation analysis, we found that the proportions of CD4, CD8 cells are significantly (*P* < 0.05) correlated with the levels of IFN-γ and TNF–α. However, the proportions of CD4, CD8, and CD56 cells are not significantly (*P* > 0.05) correlated with other clinicopathological parameters, including tumor type, IL-4, and toxicity. Due to the reason that only 15 patients were enrolled in the present phase I trial, the toxicity was summarized as either absent or present, instead of detailed toxicity reactions.

**Table 2:** Correlation analysis of the proportions of CD4, CD8, and CD56 with patient's tumor type, cytokine production, toxicity

	CD4	CD8	CD56
Tumor type	*P* = 0.309	*P* = 0.269	*P* = 0.262
Cytokine production			
IFN-γ	*P* = 0.004 (*r* = −0.669)	*P* = 0.002 (*r* = 0.722)	*P* = 0.860
TNF–α	*P* = 0.007 (*r* = −0.661)	*P* = 0.004 (*r* = −0.695)	*P* = 0.880
IL-4	*P* = 0.846	*P* = 0.319	*P* = 0.130
Toxicity	*P* = 0.913	*P* = 0.827	*P* = 0.507

### Safety analysis of TIL-based immunotherapy

Of the 15 patients for whom TILs were generated, 12 received one T-cell infusion, and three patients received two T-cell infusions. Infusion of TIL was safe and resulted in grade I and /or II toxicities, including flu-like symptoms, malaise, leucopenia and neutropenia (Table 3). Patient 1 showed a flu-like symptoms, malaise. He was administered with acetaminophen. Four patients experienced Grade 1 toxicities of leucopenia, neutropenia. No grade III/IV serious adverse events occurred in a total of 18 instances of cell injection. These toxicity gradings were assigned according to the National Cancer Institute Common Toxicity Criteria Scale 2.0.

**Table 3:** Toxicity of T-cell therapy in patients with HCC (*n* =15)

Symptoms	G0	G1	G2	G3	G4
Flu-like symptoms	14/15	1/15	—	—	—
malaise	13/15	2/15	—	—	—
leucopenia	13/15	2/15	—	—	—
neutropenia	11/15	4/15	—	—	—

### Clinical observations following infusion of TIL

After a median follow-up of 14 months, 15 patients (100%) were alive; and 12 patients (80%) showed no evidence of disease, 3 patients (patient 1,11,12) had tumor recurrence.

The time to the diagnosis of tumor recurrence following therapy ranged from 105 to 261 days (patient 1:170 days, patient 11:105 days, patient 12: 261 days)(Table 4). Patient 2 showed the longest no evidence of disease with ongoing 454 days.

**Table 4:** Patients with HCC treated with adoptively transferred TILs

Subject code	Days of T-cell infusion after the tumor tissue was obtained	Cells per infusion	Number of infusions	Clinical response	DFS (d)	OS (d)
01	26	3 × 10^9^	2	Recurrence	170	462^+^
02	26	8.5 × 10^8^	2	NED	454^+^	454^+^
03	27	3.4 × 10^8^	1	NED	438^+^	438^+^
04	31	3.1 × 10^8^	1	NED	438^+^	438^+^
05	32	8.8 × 10^8^	1	NED	431^+^	431^+^
06	31	1 × 10^9^	1	NED	431^+^	431^+^
07	25	9.0 × 10^8^	1	NED	427^+^	427^+^
08	35	8.6 × 10^8^	1	NED	427^+^	427^+^
09	22	9 × 10^8^	2	NED	425^+^	425^+^
10	28	8.2 × 10^8^	1	NED	424^+^	424^+^
11	26	2.6 × 10^9^	1	Recurrence	105	418^+^
12	26	2.5 × 10^9^	1	Recurrence	261	418^+^
13	26	9.2 × 10^8^	1	NED	404^+^	404^+^
14	27	1.8 × 10^9^	1	NED	355^+^	355^+^
15	27	10^9^	1	NED	355^+^	355^+^

+ :ongoing

NED: no evidence of disease

### Immunologic and virological responses following infusion of TIL

In the final set of our experiments, comprehensive longitudinal *ex vivo* profiling of HBV load were conducted. In addition, alpha-fetoprotein (AFP) was detected in the peripheral blood of patients with HCC who had been treated with ACT.

**Figure 1:**
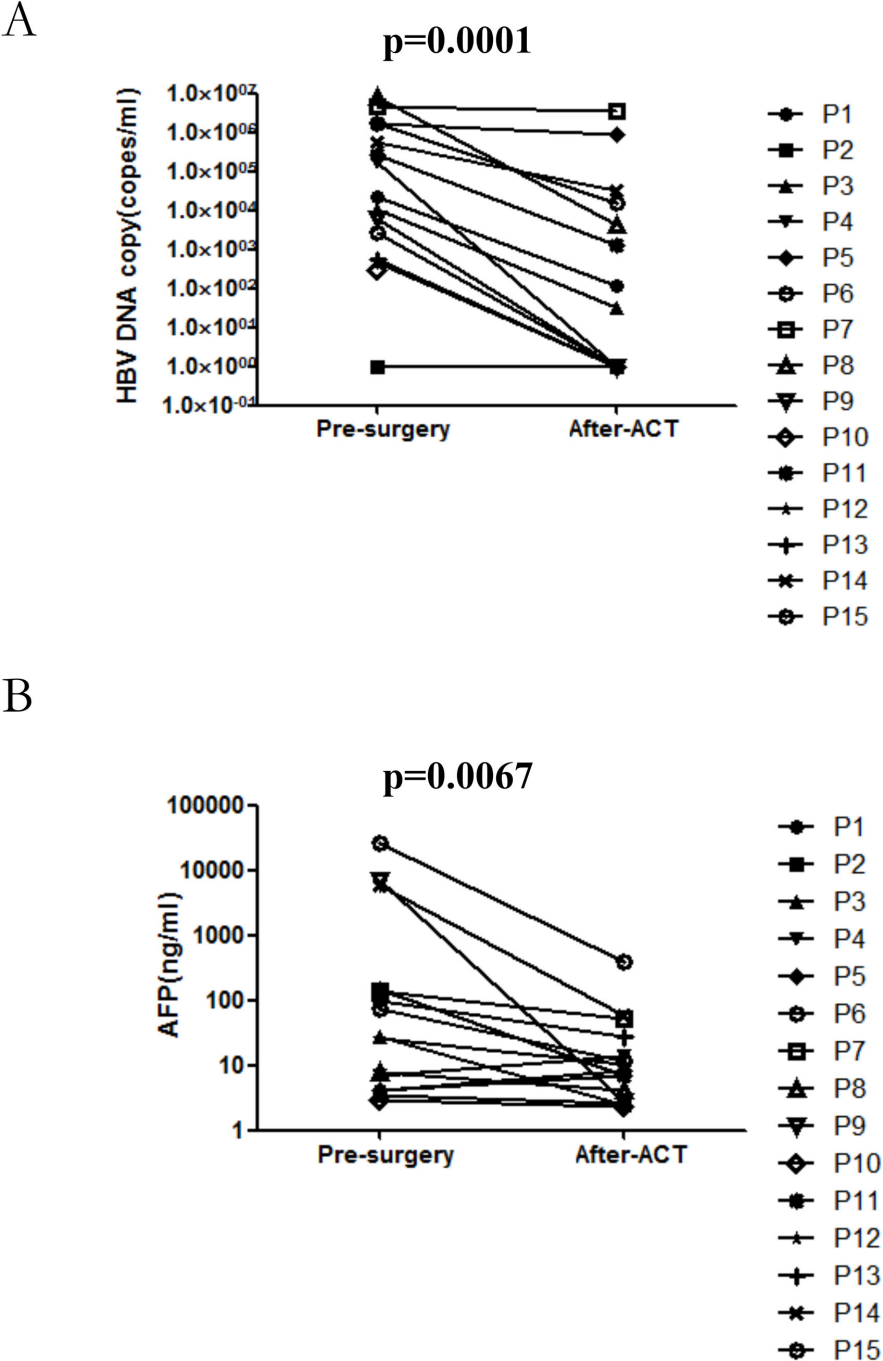
The impact of autologous TIL infusion on HBV load A. and AFP level B. Plasma samples taken prior to the surgery and after T cell infusion were assessed for HBV load and AFP level

To evaluate the plasma HBV load and AFP level, which have previously been correlated to disease status in patients with HCC [21, 22], qRT-PCR was performed on plasma samples prior to surgery and one month after infusion. As shown in Figure 1, HBV load (Figure 1A) and AFP level (Figure 1B) were either unchannged or decresed after T cell infusion. There were statistically significant changes both in HBV load (*p* = 0.0001) and in AFP (*p* = 0.0067). Additionally, the decrease of AFP and HBV level is the main cause of the reduction of the tumor burden caused by the surgical removal of the lesion.

## DISCUSSION

To our knowledge, this is the first phase I clinical trial involving ACT using *ex vivo* expanded autologous TILs after surgery as a novel treatment strategy for HCC patients. The data presented here show that infusion of autologous TILs was feasible and safe in this patient population.

Surgery is the most effective treatment for HCC, yet over 75% of HCC patients develop recurrence and/or metastasis within 5 years after surgery, which is the main cause of death for these individuals [23]. HCC possesses several characteristics that render it an attractive target for immunotherapy. For example, there is active recruitment of TILs that are able to recognize tumor-specific antigens as evidenced by their ability to lyse autologous tumor cells *ex vivo* [24]. In addition, TIL derived from HCC and expanded *ex vivo* in the presence of IL-2 displayed antitumor cytolytic activity [25].

TIL immunotherapy is a promising treatment method for cancer patients. Several researchers have demonstrated the benefit and safety of TIL immunotherapy for patients with malignancies in clinical studies [26–28]. A distinct advantage of TIL therapy is the broad nature of T-cell recognition of both defined and undefined tumor antigens against major histocompatibility complexes, in contrast to the single specificity and limited major histocompatibility complexes of the T cell receptor and chimeric antigen receptor transduction technologies.

The protocol described in this study was feasible in expanding tumor infiltrating lymphocytes from patients with primary HCC disease, and these expanded T cells have displayed high levels of IFN-γ and TNF–α expression. One of the major advantages of this procedure is that a large number of T cells can be expanded within 4 weeks. Of the 17 patients with HCC, TILs were expanded from 15 patients (88.2%), whereas there was minimal or no expansion of T cells from another 2 patients (11.8%). These *in vitro* activated and expanded polyclonal T cells are composed of both CD8^+^ T cells and CD4^+^ T cells. Adoptive transfer of TIL was safe with minimal toxicities. The data presented in this study and in previously published work demonstrate that adoptive T cell therapy displays limited toxicity [29–31] and future studies should aim to develop more strategies to provide “of-the shelf” access for such therapies for HCC patients. In recent years, adoptive immunotherapy for HCC has been limited to using autologous T cells and dendritic cells (DC) from the PBMCs of patients, including cytokine-induced killer (CIK) cells, DC vaccination, and tumor-associated antigen glypican-3 (GPC3) –derived d peptide-specific cytotoxic T lymphocytes (CTLs) [32, 33]. Previous studies by Thomas and colleagues have shown that TIL can be used for the treatment of melanoma [34]. It will therefore be important to further explore the safety of TILs for patients with HCC.

As part of the secondary objectives of the current study, our clinical follow-up analyses of the patients showed that 15 patients (100%) were alive; and 12 patients (80%) showed no evidence of disease, 3 patients had tumor recurrence. The median overall survival of patients receiving T cell therapy was remarkably longer than our institutional average during the same period. However, these preliminary observations will require confirmation in a formal phase II randomized clinical trial.

Chronic Hepatitis B virus (HBV) infection and Hepatitis C virus (HCV) infection can lead to chronic hepatitis, liver cirrhosis, and hepatocellular carcinoma (HCC). HBV is the most important etiologic agent of liver cancer globally, particularly in high-prevalence areas of liver cancer. The world's first universal HBV vaccination program was launched in Taiwan in July 1984 [35]. The prevalence of HCC among recipients of the HBV vaccine has already decreased by 70% in comparison with those in the non-vaccinated group [36]. Besides timely HBV vaccination, the earlier administration of hepatitis B immunoglobulin immediately after birth has proven to be an effective strategy to enhance the prevention of HBV infection and its related liver cancer. Before 2011, interferon (IFN)-based therapy, including dual therapy with pegylated interferon (PEG-IFN) plus ribavirin, was the standard care of HCV patients in most part of the world. Since 2011, several effective direct antiviral agents (DAAs) have been approved by the U.S. Food and Drug Administration for the treatment of HCC. Several trials have examined the impact of antiviral agents on HCV-related HCC patients [37–40]. IFN-based therapy may decrease HCC incidence in HCV cirrhotic patients after *a* > 5-year follow-up; improve liver reserve; decrease HCC recurrence rate, and increase survival rate in HCV-related HCC patients after curative HCC therapy.

Taken together, the results of the current study demonstrate that adoptive immunotherapy with TILs is safe and feasible in patients with HCC. These findings provide an important platform for future lager scale clinical trials with TIL–based immunotherapy in patients with HCC.

## MATERIALS AND METHODS

### Study design

This study was a phase I clinical trial assessing the use of TIL for adoptive cell therapy for patients with HCC. The study was approved by the Ethics Committee on Clinical Investigation of the Cancer Center of Sun Yat-sen University (Guangdong, China) and is registered with Clinical Trials.gov (NCT01462903). Written informed consent was obtained from all participating individuals. The trial was carried out in accordance with the Helsinki declaration on experimentation on human subjects. The primary endpoint of this trial was to evaluate the safety of this therapy. The secondary endpoint was to investigate the immunological response and clinical outcome.

### Patient eligibility

Patients with primary HCC in the Department of Hepatobiliary Oncology, Sun Yat-sen University Cancer Center from January 2014 to May 2014 were enrolled for this study. The eligibility criterias for patients participating in the clinical trial were as follows: (1) Patients were planned for radical resection; (2) Evaluation of clinical response in these patients was possible; (3) Child-Pugh scores were ≤ 9; (4) Eastern Cooperative Oncology Group (ECOG) performance status was 0–2; (5) Expected survival was at least 3 months; (6) Age of the patients was between 20–80 years; (7) Adequate bone marrow, cardiac, pulmonary and renal functions were present, including a white blood cell count of > 2000/mm^3^, platelet count > 75000/mm^3^, total bilirubin < 1.5 times the institutional normal upper limits, creatinine < 1.5 times the institutional normal upper limits, and AST/ALT/ALP < 2.5 times the institutional normal upper limits; (8) Patients who had provided informed consent. The exclusion criterias of patients participating in the clinical trial were as follows: (1) Previous treatment with IL-12; (2) Pregnant or breastfeeding women, due to the potentially dangerous effects of the preparative chemotherapy on the fetus or infant; (3) Patients with active systemic infections, coagulation disorders or other major medical illnesses of the cardiovascular, respiratory or immune system, myocardial infarction, cardiac arrhythmias, obstructive or restrictive pulmonary disease; (4) Patients with any form of primary immunodeficiency (such as severe combined immunodeficiency disease); (5) Patients with concurrent opportunistic infections, as the experimental treatment being evaluated in this protocol depends on an intact immune system. Patients who have decreased immune competence may be less responsive to the experimental treatment and more susceptible to its toxicities; (6) Patients undergoing concurrent systemic steroid therapy; (7) Patients with concurrent autoimmune diseases. Patient characteristics are detailed in Table 5. After patient enrollment and clinical verification of disease status, surgery to obtain tumor tissue was performed. Tumor tissue was used to expand TIL and to generate tumor lysate. As soon as successful TIL expansion was confirmed, patients received T cell infusion once or twice at two day intervals depending on the cell numbers obtained.

**Table 5:** Characteristics of patients with HCC enrolled in the phase I clinical trial

Subject code	Age	Sex	ECOG	Hepatic virus infection	Cirrhosis	Prior therapy
01	66	Male	1	HBV	No	Surgery
02	37	Male	0	HBV	No	Surgery
03	33	Male	0	HBV	Yes	Surgery
04	51	Male	0	HBV	Yes	Surgery
05	59	Male	1	HBV	Yes	Surgery
06	54	Male	0	HBV	Yes	Surgery
07	56	Male	0	HBV	Yes	Surgery
08	56	Male	1	HBV	No	Surgery
09	52	Male	0	HBV	No	Surgery
10	31	Male	0	HBV	Yes	Surgery
11	40	Male	0	HBV	Yes	Surgery
12	57	Male	0	HBV	No	Surgery
13	41	Female	0	HBV	No	Surgery
14	35	Male	0	HBV	Yes	Surgery
15	33	Male	0	HBV	Yes	Surgery

### Generation and characterization of tumor-infiltrating lymphocytes

Bulk TILs were obtained from biopsied adjacent-tumor tissues and expanded using an REP under conditions in accordance with current good manufacturing practices (GMP) in the Biotherapy Center at Sun Yat-sen University Cancer Center. Tumor specimens were sliced with a scalpel into small pieces of approximately 2–3 mm^3^ in size. Enzymatic digestion of the pieces was performed for 1.5 to 2 hours at 37°C with media containing collagenase IV (0.1 μg/ml) (Sigma-Aldrich, Saint Louis, USA). The obtained single-cell suspension was passed through a cell strainer, purified with a Ficoll gradient to collect TILs, washed twice with phosphate buffered saline (PBS). The TILs were then transferred to a 12-well plate at a concentration of 1.0 × 10^6^ cells/mL in X-VIVO-15 medium (Lonza, Walkersville, USA) containing 10% human AB serum and recombinant human IL-2 (2000 IU/ml, Four Rings Bio-Pharmaceutiacl Co, Beijing, China) for cell expansion. Cell expansion generally required approximately 10 to 14 days. Short-term cultured TILs were then immediately cryopreserved or used directly for further large-scale cell activation and expansion. A total of 30–60 × 10^6^ TILs were activated and expanded to treatment levels by REP using an anti-CD3 antibody (OKT-3, 30 ng/ml, BioHermes, Wuxi, China), 4000 IU/mL rhIL-2, and irradiated feeder cells as previously described [41].

### Adoptive cell transfer

**Figure 2:**
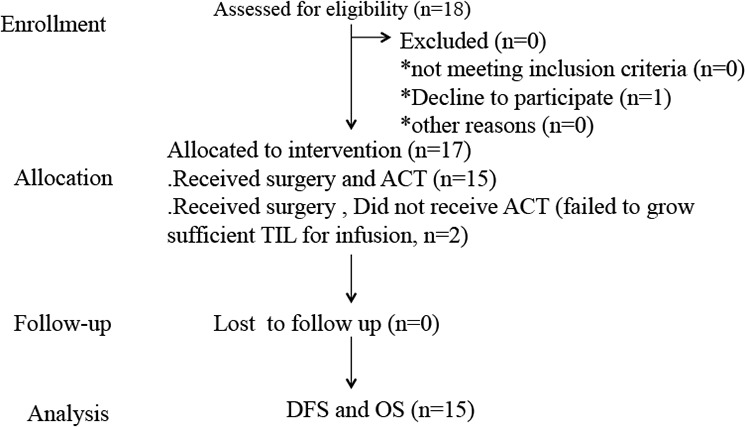
Phase I clinical trial profile using TIL for HCC patients

The T cell expansion of each patient is shown in Table 4. Patients who had successful T cell expansion received 1–2 infusions of 0.3 × 10^9^ to 3 × 10^9^ T cells per infusion. None of the patients were administrated with IL-2. Peripheral blood samples were harvested prior to the first infusion and up to 1 month post-infusion to assess the impact of T cell infusion on Peripheral blood cell count and to monitor plasma HBV load. Patients were monitored for tolerability and safety following T-cell infusion. Follow-up was in our outpatient department, and involved clinical and laboratory examinations 1 month after first infusion, every 3 months for the first 2 years, every 6 months during the third to fifth years, and annually for an additional 5 years or until death, whichever occurred first. MRI or computed tomographic scanning were conducted on patients to assess baseline tumor load prior to infusion and 1, 3, and 6 months after first infusion. Treatment responses were determined on the basis of WHO criteria and/or Response Evaluation Criteria in Solid Tumors (RECIST) (Figure 2).

### Immunologic monitoring assays

TILs were collected and stained with the following antibodies: anti-CD3 (Pcy5), anti-CD4 (PE), anti-CD8 (ECD), and anti-CD56 (APC) (Biolegend, CA).CD27 and CD28 surface expression was further tested after incubating TIL in complete medium without rhIL-2 for 48 hours. Isotype-matching antibodies were used as controls. After incubation at 4°C for 30 min with the above coupled antibodies and being washed with PBS, TILs were fixed with 4% paraformaldehyde and analyzed using CytomicsFC500 Flow Cytometry (Beckman Coulter Inc., Fullerton, CA). Data analysis was performed using the CXP Analysis software (Beckman Coulter). For cytokine production assays, TILs were stimulated with paraformaldehyde (PMA) (Sigma, 50 ng/ml) and ionomycin (Sigma, 500 ng/ml) for 4 h in the presence of Brefeldin A (Biolegend; 10 mg/ml). Cells were then collected, washed and fixed with 4% PMA for 5 min at room temperature (RT) and permeabilized with permeabilization buffer (eBioscience, San Diego, CA) for 10 min at RT. Cells were then labeled with anti-IFN-γ (APC), anti-TNF-α (FITC), and anti-IL-4 (APC) and analyzed by flow cytometer.

### HBV DNA quantification

All of the patients were tested for HBV DNA by means of quantitative real-time PCR using a COBAS AmpliPrep/COBAS TaqMan automated system (HBV Test, Roche Diagnostics, Bael, Switzerland). The HBV load was compared pre-surgery *vs*. after-ACT for each patient. Disease-free survival (DFS) was calculated from the time of surgery to the first detectable recurrence or progression, whereas overall survival (OS) was calculated from the time of surgery to the time of last follow-up.

### Statistical analysis

The HBV load and AFP from 15 patients who were pre-surgery and post-ACT were analyzed by paired *t*-test.
